# The role of prefrontal-hippocampal functional connectivity in schizophrenia-related cognitive dysfunction and the thalamic ventral midline involvement: *in vivo* and *silico* evidence

**DOI:** 10.3389/fnins.2025.1653828

**Published:** 2025-11-10

**Authors:** Anastasija Černousova, Enrico Patrono

**Affiliations:** Center for Advanced Behavioral Research (CABR), School of Psychology, University of New York in Prague (UNYP), Prague, Czechia

**Keywords:** schizophrenia, prefrontal cortex, hippocampus, thalamus, interaction, functional connectivity, *in vivo*/*in silico* models

## Abstract

Schizophrenia (SCZ) is a multiform psychiatric disorder in which impairments of high-order cognitive abilities, such as flexibility, working memory, and decision-making, are considered onset markers. These deficits are associated with dysfunctions in the prefrontal cortex (PFC) and hippocampus (HPC), two brain regions that play crucial roles in higher-order cognitive processes. While the roles of the PFC and HPC in SCZ have been widely studied, the interaction between these regions and their contributions to the observed cognitive deficits, in conjunction with other intermediate structures, refMRI connectivity as a biomarker main poorly understood. This paper primarily aims to create a hypothesis-generating framework in the context of PFC-HPC altered communication and intermediate structures that may contribute to cognitive impairments in psychosis-related conditions. Here, we present several testable hypotheses concerning the role of specific actors (e.g., GABAergic Parvalbumin-positive interneurons, thalamic calcium signaling channels) in the PFC-HPC connectivity. By presenting evidence from *in vivo* (animal models and human studies) and *in silico* studies (examining functional connectivity), we desire to reach computational and translational researchers, with the aim of stimulating further planning for new experimental methodologies, both computational and translational, that can provide a broad framework for a more nuanced understanding of maladaptive brain communication in psychosis.

## 1 Introduction

Schizophrenia (SCZ) is a multiform psychiatric disorder that alters various aspects of an individual’s life, like how reality is perceived, how emotions are regulated, and how social interactions work out. The SCZ symptoms are typically grouped into two broad categories: the positive symptoms and the negative symptoms. The positive symptoms usually entail hallucinations and delusions, while the negative symptoms predominantly describe social withdrawal, emotional dysregulation, and anhedonia (Kulhara and Gupta, 2010). Additionally, SCZ presents with cognitive deficits, specifically in high-order cognitive abilities within the executive function context (McCutcheon et al., 2023), such as working memory, decision-making, and flexibility. These deficits interfere with day-to-day functioning, ranging from problem-solving to social interactions. However, what makes these issues and impairments specifically challenging is the fact that they tend to persist regardless of treatment, as the antipsychotic medication does not have a direct impact on the cognitive deficits (Mosolov and Yaltonskaya, 2022). Moreover, SCZ-like cognitive impairments have been recently valued as endophenotypic markers of the onset of the pathology (Greenwood et al., 2019). Notably, it has been suggested that the cognitive deficits may arise from a primary impairment in the filtering or “gating” of sensory information, with an incapacity to filter out irrelevant external stimuli, possibly leading to the psychotic symptoms (e.g., hallucinations, delusions) and cognitive disorganization (Braff and Light, 2005). Therefore, it is important to explore alternative hypotheses underlying SCZ-like cognitive impairments. This study will focus particularly on the altered flexibility and poor decision-making commonly observed in SCZ.

Furthermore, an increasing number of findings suggest that these higher-order inabilities may stem from “disrupted communication” between two critical brain regions: the prefrontal cortex (PFC) and the hippocampus (HPC), which are essential for higher-order cognitive processes (Aleman, 2014; Szczurowska et al., 2017; Ferguson and Gao, 2018; Peterson et al., 2023; Patrono et al., 2023, 2025). Over the last two decades, accumulating morphological, electrophysiological, and functional evidence has shown that disruption of the PFC-HPC often alters their dynamic interaction, depending on various task demands (Sigurdsson and Duvarci, 2016). These studies significantly advanced our understanding of how our brain operates as a large-scale network to support diverse sensory, cognitive, and behavioral functions. In addition, it has been hypothesized that SCZ-like cognitive inabilities could be rooted in the discoordination of activity within neuronal networks (discoordination hypotheses, Szczurowska et al., 2017), highlighting the importance of coordination in neuronal firing for information processing, and coordinated activation of cell assemblies (coalitions of co-active cells that constitute the minimal functional units of information encoding and processing in the brain). Thus, synchronization of neuronal activity on timescales ranging from tens of milliseconds to seconds is crucial, and any desynchronization of the activity of such cell assemblies is hypothesized to cause impaired information processing in SCZ.

Furthermore, another convincing hypothesis explaining SCZ-like cognitive impairment is related to an imbalance in the excitatory/inhibitory (E/I) ratio. The E/I ratio refers to the balanced state of singular entities’ overall excitatory and inhibitory levels at the single-cell and global circuit levels (Ferguson and Gao, 2018). Sohal and Rubenstein (2019) clarified the multidimensional concept of this balance, stating that excitatory and inhibitory signals originate from multiple sources and can act on different targets, such as the PFC and the HPC. This idea has sparked discussions on the possibility of enhancing specific inhibitory activity in those regions to restore the E/I balance, resynchronize the coordination of GABAergic cell assemblies, and preserve cognitive abilities (Patrono et al., 2023). The PFC, particularly the dorsolateral PFC (dlPFC), is historically considered a brain area associated with executive control functions such as task switching and task-set reconfiguration, prevention of interference, inhibition, planning, and working memory (Badre and Wagner, 2004; Hart et al., 2013; Brunoni and Vanderhasselt, 2014). On the other hand, the HPC plays a crucial role in forming and retrieving memories, especially episodic memories (explicit memories of everyday events), spatial navigation, emotional regulation, and context processing, all of which are impaired in SCZ (Wiltgen et al., 2010; Burgess et al., 2002; Pronier et al., 2023). Recent works from our group showed that optogenetic stimulations of GABAergic parvalbumin-positive interneurons (PV+) at specific frequencies in the medial PFC (mPFC) and the ventral HPC (vHPC) rescued previously MK801-altered behavioral flexibility (perceptual; Patrono et al., 2023; and navigational; Patrono et al., 2025), suggesting that MK801-induced N-Methyl-D-Aspartate (NMDA) hypofunction was balanced by increased PV + inhibitory activity. Thus, it becomes evident that a disruption of inter-regional coordination is a crucial pathophysiological mechanism in several psychiatric disorders, including SCZ. At the same time, it has been recently advocated that various subcortical structures of the thalamic ventral midline, including the nucleus reuniens (nRE), the basal ganglia, and the subthalamic nuclei, mediate such communication (Colgin, 2011; Shapiro et al., 2014; Shin and Jadhav, 2016; Ferraris et al., 2021; Schlecht et al., 2022; Panzer et al., 2024). Another objective of this work is to summarize recent *in vivo* findings reporting the increasing evidence of a thalamic involvement in the PFC-HPC “crosstalk,” which is altered in SCZ (Table 1).

**Table 1:** Summary of the findings for the animal models of SCZ.

Authors	Methods/Purpose	Research design	Target population	Major findings
Speers et al. (2021)	MIA	Poly I:C treatment in pregnant rats; in-vivo electrophysiology LFPs in dorsal HPC	Poly I:C treated male rat progeny	Phase precession (the time of firing of action potentials by individual neurons occurs progressively earlier) and Θ sequences are disrupted in a MIA model of SCZ risk
Wang et al. (2024)	PFC-HPC connectivity	amyloid beta (Aβ) rat model; in-vivo electrophysiology LFPs in PFC-HPC ensembles	rats	Coordinated PFC-HPC sequences showed PFC-dominant prediction of goal locations during successful memory retrieval.
Condon (2024)	Kv3	extracellular recordings in the PFC and HPC of awake, head-fixed Kv3.1KO or Kv3.3KO mice	mice	Impaired synchrony between the mPFC and HPC was found in the low frequency bands, and an increased synchrony in the high frequency bands in Kv3.1KO mice and Kv3.3KO mice.
Featherstone et al. (2022)	CaMKIIα	CaMKIIαKO mice; EEG; anxiety-like behavioral testing	mice	CaMKIIαKO mice were hypoactive and less anxious; displayed substantial impairments in long-term memory; and showed increased amplitude of Θ frequency oscillations.
Tseng et al. (2009)	NVHL	Neonatal and adult ibotenic lesions of the vHPC; behavioral measurements	rats	Neonatally induced lesioned rats were hyperresponsive to stress in adulthood compared to controls, suggesting that the neonatal vHPC lesion may affect the functional development of the mPFC
O'Donnell (2012)	NVHL	Neonatal and adult ibotenic lesions of the vHPC; behavioral measurements	rats	One of the principal elements affected in NVHL rats is the dopamine modulation of PFC cortical interneurons
Druart et al. (2021)	E/I ratio in PFC	in utero electroporation C4-overexpressing (C4-OE) mice in mPFC; NMDA receptor hypofunction; GABA-related GAD67 expression	mice	Found reduced GLUergic input to pyramidal cells of juvenile and adult, but not of newborn (C4-OE) mice, together with decreased spine density; evidence for NMDA receptor hypofunction; lower GAD67 expression, and decreased intrinsic excitability in PV+ interneurons

The table summarizes the significant conclusions related to SCZ hallmarks using pharmacological and genetic animal models. All the models listed produced specific SCZ hallmarks such as desynchronization of Θ waves in PFC and HPC. MIA, maternal immune activation; Poly I:C, Polyinosinic:polycytidylic Acid; LFPs, local field potentials; Kv3.1KO, potassium voltage-gated channel 3.1 knock out; Kv3.3 KO, potassium voltage-gated channel 3.3 knock out; CaMKIIα, calcium/calmodulin-dependent protein kinase II; CaMKIIαKO, calcium/calmodulin-dependent protein kinase II knock out; EEG, electroencephalogram; NMDA, N-methyl D-aspartate; GABA, gamma aminobutyric acid; GAD67, glutamate decarboxylase 67; Pv+, parvalbumin positive.

While the PFC-HPC “crosstalk” disruption, with the potential involvement of subcortical ventral midline structures, has been recently suggested in humans and animal models of psychiatric conditions, *in silico* studies, specifically those involving computational modeling, can help simulate those PFC-HPC interactions, aiding in the exploration of how factors such as neurotransmitter imbalances or synaptic dysfunction may shape cognition, for a more well-rounded argumentation. One helpful way to interpret the evidence across various studies is through the functional connectivity hypothesis (Del Fabro et al., 2021; Katsumi et al., 2021), which proposes that cognitive deficits in SCZ may not originate solely from the structural brain abnormalities, but from a network-level disruption in the coordinated activity between the regions (Sendi et al., 2021). Therefore, by reviewing the evidence from *in vivo* and silico studies regarding the breakdown of the PFC-HPC communication network and its intermediary structures, we aim to provide testable predictions that could validate translational approaches. It becomes clearer what role they play in SCZ-related cognitive dysfunctions. Understanding the mechanisms underlying PFC-HPC dysfunction in SCZ not only helps us further deepen our knowledge of SCZ but also provides a broader perspective on how disrupted neural circuits induce cognitive impairments in psychosis-related disorders.

### 1.1 Methods

This narrative review synthesized literature from PubMed, Web of Science, and Google Scholar using search terms including “schizophrenia,” “prefrontal cortex,” “hippocampus,” “nucleus reuniens,” “cognitive flexibility,” “decision-making,” “functional connectivity,” and “computational models” for the period 2000–2025. We prioritized peer-reviewed empirical studies, meta-analyses, and computational modeling papers while excluding case reports and non-English publications.

## 2 Overview of schizophrenia/psychosis-related disorders

The Diagnostic and Statistical Manual of Mental Disorders (DSM-5) defines SCZ as a psychiatric disorder characterized by significant disturbance in thought processes, emotional regulation, and behavior (APA, 2013). SCZ is distinct in its persistent and severe cognitive dysfunctions, which target flexibility and decision-making, but also social and emotional regulation, contributing to its debilitating impact on daily functioning. Importantly, the cognitive deficits in SCZ are more pervasive and resistant to treatment, underscoring the need for further research (Lawrence et al., 2024). Based on the early words of Emil Kraepelin, SCZ is likened to “an orchestra without a conductor” (Bar and Ebert, 2010), suggesting that while some functions may seem normal, a particular key “conductor” is missing, keeping everything in harmony.

### 2.1 Symptom clusters of SCZ

The primary symptoms of SCZ can be categorized into positive and negative symptoms. The positive symptoms are delusions, hallucinations, disorganized thinking, and abnormal body movements. The negative symptoms primarily involve reduced emotional expression and a lack of motivation (APA, 2013). A growing body of research is investigating alternative origins or contributing factors to SCZ and other psychotic disorders (Walther et al., 2021; Dienel et al., 2023).

While positive and negative symptoms are the main clusters of symptoms, recent evidence recognizes cognitive symptoms as a potential “third cluster” (Moura et al., 2021; Wu et al., 2021), as they have a high and direct impact on an individual’s ability to process information, adapt to new situations, and make goal-oriented decisions. Cognitive symptoms can have a crucial role in the differential diagnosis with respect to other psychotic disorders. Therefore, cognitive deficits are increasingly discussed as key features that differentiate and unite the aetiopathology of psychotic disorders (Lawrence et al., 2024).

### 2.2 State-dependent network dynamics in SCZ

In recent years, extensive research has confirmed that SCZ involves distinct functional brain network dysfunctions both at rest and during specific tasks. Findings in both areas often correlate with clinical symptoms, including positive, negative, and disorganized symptoms, as well as social cognitive deficits. These abnormalities can be observed in networks such as the default mode network (DMN), the frontoparietal, central executive network (CEN), and the sensorimotor network (SMN), highlighting how disruptions in intrinsic brain communication contribute to a range of impairments in the disorder (Nekovarova et al., 2014; Sheffield and Barch, 2016; Jimenez et al., 2019). At rest, studies using resting-state functional magnetic resonance imaging (rs-fMRI) showed abnormalities in intrinsic functional connectivity within and between key brain networks, including the DMN, CEN, and salience network (SN). The consistent findings in resting-state networks, particularly the DMN, have led to the idea that rs-fMRI could serve as a biomarker for schizophrenia, aiding in early diagnosis and targeted treatment development. Furthermore, research supports the triple network hypothesis, which posits that dysfunctions in these interconnected networks are central to schizophrenia pathology (Nekovarova et al., 2014). In particular, the triple network hypothesis represents a unified mechanism underlying a wide array of symptom domains in SCZ, including deficits in self (self-awareness and self-representation) and theory of mind dysfunctions, as well as the traditional positive, negative, and cognitive domains.

Conversely, during awake, task-based conditions, SCZ individuals often exhibit different patterns of neural activation and connectivity compared to healthy controls. Dysfunctions are observed in networks, such as the visual network and the sensorimotor network, during task performance. Furthermore, task-based studies show a link between disrupted visual network connectivity and deficits in social cognition, a key area of impairment in SCZ (Sheffield and Barch, 2016; Jimenez et al., 2019). Moreover, task-fMRI data showed that reduced suppression of the DMN and CEN in SCZ patients during cognitive tasks, suggesting that disrupted DMN activity is only present in SCZ patients with impaired cognitive function (Zhou et al., 2016; Jia et al., 2020).

Finally, the observed functional connectivity abnormalities in both resting-state and task-based conditions suggest that these disruptions may reflect shared underlying mechanisms.

### 2.3 Cognitive impairments as a core feature

Cognitive impairments in SCZ not only persist beyond symptomatic episodes but also, oftentimes, precede the onset of full psychosis and remain stable throughout the disorder. Øie et al. (2021) have demonstrated that individuals at high risk for SCZ or other psychotic disorders already exhibited deficits in various cognitive functions, such as working memory or executive functioning, before experiencing their first psychotic episode, independently of other SCZ cluster symptoms, thus suggesting that cognitive dysfunction is not simply a consequence of psychosis. Hence, cognitive impairments are considered a potential early biomarker of SCZ and psychotic disorders’ pathology (Øie et al., 2021). Furthermore, it has been demonstrated that SCZ-related cognitive deficits extend across multiple domains, resulting in significant difficulties in an individual’s daily life (Fu et al., 2020, 2021). For example, cognitive flexibility is also impaired in individuals with SCZ, leading to rigid thought patterns and poor problem-solving skills (Zhu et al., 2021). In addition, other cognitive deficits related to executive functions (working memory, planning, inhibitory control, and attentional control) are impaired (Zhu et al., 2021). Such impairments constitute a significant barrier to independent functioning, as they affect occupational performance, social relationships, and daily life activities. Another important aspect is related to the onset of SCZ. Before apparent symptoms emerge, there can be years of subtle cognitive and social changes, including poor academic performance, social withdrawal, and unusual thoughts and behaviors. The typical age is late teens to early 30s, with symptoms appearing earlier in men (late teens to early 20s) and later in women (late 20s to early 30s). Onset before age 18 is considered early-onset, and onset before age 13 is sporadic (McGrath et al., 2008; Hollis and Rapoport, 2008).

In general, antipsychotic medications can effectively reduce positive symptoms by modulating dopamine or serotonin receptor activity. For example, second-generation antipsychotics are serotonin–dopamine antagonists and are also known as atypical antipsychotics (risperidone, olanzapine, quetiapine, ziprasidone, aripiprazole, etc.). In clearly defined treatment-resistant SCZ, clozapine generally outperformed other antipsychotics, especially when dosed appropriately (Asenjo Lobos et al., 2010). However, the heterogeneity of antipsychotic response across individuals and key symptom domains, such as the considerable degree of nonresponse/treatment resistance in multi-episode patients, and the adverse effect potential of antipsychotics, are significant limitations, underscoring the need to develop new medications for the SCZ treatment of SCZ. Such antipsychotics offer minimal, if any, improvement in cognitive dysfunctions. This suggests that cognitive impairments arise from distinct neurobiological mechanisms, separate from the dopaminergic dysfunction associated with psychosis (Buck et al., 2022; de Bartolomeis et al., 2022; Howes and Shatalina, 2022). Therefore, since cognitive impairment is not resolved with standard pharmacological treatments, it becomes essential to research and expand the understanding of the altered neural basis involved in SCZ-like cognitive dysfunctions. Given the severity, persistence, and resistance to treatment of cognitive impairments in SCZ, identifying and focusing on their neural mechanisms is crucial for advancing research on this topic. Various studies suggest that cognitive dysfunctions are closely tied to aberrant neural oscillations in the PFC-HPC circuit (Sigurdsson and Duvarci, 2016; Shing et al., 2022). As previously mentioned, the PFC is important for executive control, decision-making, working memory, and flexibility, while the HPC plays a crucial role in encoding and retrieving contextual information. Several studies have examined the separate impairments of either the PFC or HPC and their effects on SCZ. However, a growing number of studies are investigating PFC-HPC connectivity and how they might communicate with each other, suggesting that a breakdown in PFC-HPC crosstalk may underlie rigid thought patterns, poor problem-solving abilities, and disorganized cognition (Sigurdsson and Duvarci, 2016).

To better understand and frame the cognitive impairments observed in SCZ, it may be beneficial to review the domain-general and domain-specific cognitive processes that are involved (McCutcheon et al., 2023; Réthelyi et al., 2012). In SCZ, both domain-general and domain-specific cognitive deficits are observed. Domain-general deficits, like slower processing speed, are widespread across various cognitive domains. However, specific areas, such as working memory and episodic memory, also show significant impairments, suggesting domain-specific deficits. The concept of domain-specific processes refers to cognitive skills related to specific types of information, such as language, social cognition, executive functions, and spatial awareness. These skills are associated with corresponding brain regions, including the PFC and the HPC. The domain-general capacities and functionings are also typically described as an “orchestrated” working, as they combine and integrate different systems and approaches for unified thinking and behavior (Li et al., 2014). However, there is an interplay between general and specific cognitive deficits. The general deficit can impact performance in specific domains, while specific deficits in certain areas may also contribute to overall cognitive impairment (McCutcheon et al., 2023). Such frameworks may help in understanding SCZ, where both types of processes are usually disrupted. For example, suppose there are impairments in domain-general functioning, such as attention or general cognitive control. In that case, it can, in turn, compromise the integration of information from domain-specific systems, further worsening the functional impact. Examining SCZ through this approach highlights that cognitive dysfunctions in these disorders may reflect not just isolated deficits, but a more systematic breakdown in the coordination of cognitive operations.

By looking more closely into the PFC-HPC functional connectivity, one may offer a valuable perspective into the network-level alterations that underlie the cognitive dysfunctions in SCZ. An outlook like this examines research and evidence beyond isolated brain regions. It considers how breakdowns in communication between key areas may contribute to impairments in processes such as decision-making, memory, and cognitive flexibility. The following section of this paper will explore and evaluate the empirical evidence gathered from *in vivo* and silico studies, further drawing on evidence to support the disrupted PFC-HPC interactions and their relationship to higher-order cognitive deficits in SCZ.

## 3 *In vivo* behavioral imaging

Various imaging methods, such as fMRI and electroencephalography (EEG), aid research by observing how different brain regions communicate and interact in real-time, both in normal and pathological cases. Advantageously, when these functional techniques are paired with behavioral assessments, these tools help uncover specific patterns of disrupted connectivity that may underlie the SCZ-like cognitive disabilities. However, it is essential to note that humans and other animal species used as models differ in brain organization due to variations in cortical amount, size, complexity, and connections, as well as differences in the developmental timing of neuron generation and structural complexity. These variations enable specialized behaviors, such as human language and social cognition, compared to other mammals like monkeys and rodents, which show different regional specializations and neural densities. For instance, the human brain has expanded certain cortical regions, such as the temporal cortex, and increased synaptic connectivity, whereas other species may exhibit different patterns of development and functional emphasis (Eichert et al., 2020).

### 3.1 Functional magnetic resonance imaging

Among various available neuroimaging techniques, fMRI is one of the most commonly used methods for examining and investigating brain function. It primarily focuses on connectivity and structure, particularly in pathological cases, such as SCZ, or assessment cases. The blood oxygen level-dependent (BOLD) signals directly showcase an indirect yet dynamic measure of neural activity over time, which is essential for exploring how the PFC and the HPC interact, and more importantly, whether this interaction is altered or disrupted in SCZ individuals. In recent years, fMRI studies have shown diminished PFC connectivity with brain regions regulating dopamine (DA), such as the substantia nigra and striatum. This impairment may be linked to PFC hypofunction and contribute to the dysfunction of these DA-regulating regions (Yoon et al., 2023; Zeng et al., 2022), which are involved in processing motivation and effort. These findings may also explain why many people with SCZ experience diminished motivation or drive. The dlPFC is historically considered a brain area associated with domain-general executive control functions, such as task switching, task-set reconfiguration, interference prevention, inhibition, planning, and working memory (Badre and Wagner, 2004; Hart et al., 2013; Brunoni and Vanderhasselt, 2014). Working memory is the dynamic ability to hold and manipulate information while performing different cognitive tasks, and it is involved in the transition from short-term to long-term storage (Friedman and Robbins, 2021). In combination with the HPC, which plays a crucial role in forming and retrieving memories—specifically episodic memories—working memory, along with flexibility and decision-making, form the main framework to study the PFC-HPC connectivity in SCZ. A complementary study focused on working memory in SCZ individuals and those with major depressive disorder (MDD). The results revealed that individuals with SCZ exhibit reduced activity in the mPFC, frontolateral PFC (flPFC), and middle frontal gyrus (Hwang et al., 2021; Wang C. et al., 2021; Wang X. et al., 2021; Wang Y. et al., 2021). The study also observed an interesting overactivation of the default mode network (DMN) during working memory tasks in SCZ patients. This suggests that these individuals struggle to suppress internal noise, as the DMN is typically deactivated during externally focused tasks (Spreng et al., 2014). These results further support the concept that modifications in PFC functioning can impair cognitive functioning, thereby making it more challenging to engage in flexible thinking and decision-making. This further reiterates the idea of network-level dysfunctions, as impaired PFC connectivity affects systems involved in top-down cognitive processing (Chari et al., 2019).

One established method to investigate the domain-generality vs. domain-specificity of PFC-HPC functional connectivity in SCZ is called “seed-based functional connectivity.” It is employed to identify the resting-state networks and to provide a direct way to examine the regions with robust functional connectivity within a “seed brain region” (a specific region of interest corresponding to a specific brain structure). A recent fMRI study employed this methodology to investigate the connectivity abnormalities in SCZ in the anterior–posterior subregions of the HPC during episodic memory (Dugré et al., 2021). It was found that there was a significant reduction in connectivity between the posterior HPC and the PFC regions. Still, an increased connectivity between the anterior HPC and the anterior temporal regions, thus suggesting functional differences between the anterior and posterior portions of the HPC (Lyttle et al., 2013), which reflects the deepening of the understanding of the role of both single loci of brain regions and their connections with other cognitive-functioning loci of the brain.

Another study investigated the changes in the HPC in first-episode psychosis (FEP) patients (Alho et al., 2023). The participants were instructed to watch scenes from the movie titled Alice in Wonderland, which provided high ecological validity for the methods of observing functional brain dynamics. During their initial scans, FEP patients exhibited stronger HPC network connectivity compared to the control group, particularly in circuit areas associated with memory, decision-making, and cognitive control, such as the thalamus, striatum, and orbitofrontal cortex (ORB). Surprisingly, this hyperconnectivity decreased in FEP patients after 1 year. In contrast, this remained unchanged in controls, suggesting a fascinating finding that these early-stage changes in connectivity may evolve, directly illustrating the dynamic shifts in the disorder as it progresses, specifically in HPC-related circuits. Additionally, the scans showed a correlation between the worsening of the HPC-centered network and an increase in delusions and other positive symptoms in FEP patients. This further implies that excessive connectivity in the HPC network might contribute to positive symptoms in early psychosis, linking this study not only to SCZ patients but also to other psychotic disorders.

Hippocampal dysfunction in SCZ is more than just memory loss. The HPC also has a crucial role in spatial navigation, emotional regulation, and context processing, all of which are impaired in SCZ and other psychosis-related disorders. Moreover, the HPC can be divided into dorsal (dHPC) and ventral (vHPC) regions. The dHPC corresponds to the posterior HPC in primates, performing primarily cognitive functions. The vHPC (anterior in primates) is associated with stress, emotion, and affect. Notably, gene expression in the dHPC correlates with that in cortical regions involved in information processing. In contrast, genes expressed in the vHPC correlate with regions involved in emotion and stress (amygdala and hypothalamus) (Fanselow and Dong, 2010). Furthermore, studies on animal models of SCZ have reported that lesions to the dHPC in rats impair performance on spatial memory tasks. In contrast, vHPC lesions often did not affect spatial learning (Moser and Moser, 1998; Bannerman et al., 2004). In humans, dissociations between anterior (vHPC in rodents) and posterior (dHPC in rodents) hippocampal functions have also been observed with fMRI (Dugré et al., 2021; Strange et al., 2014). Functional dissociations between the human anterior and posterior HPC have been attributed to the processing of novel versus repeated stimuli, emotional versus non-emotional material, and encoding versus retrieval (Spaniol et al., 2009). These results support a model of “tonic hyperactivity” of the anterior hippocampus (vHPC) in patients compared to healthy controls (Grace, 2012). According to this view, increased activity in the anterior HPC disinhibits the ventral tegmental area in the basal ganglia, contributing to increased DAergic activity and disordered cognition in SCZ. Conversely, another interesting finding showed that anterior HPC activity increased during novelty detection in healthy controls, but was reduced in unmedicated individuals with SCZ, not in medicated individuals (Tamminga et al., 2012). This suggests that the anterior HPC regulates access to newly acquired information, while the posterior HPC is activated to consolidate and retrieve that information. This means that the flexible and continuous acquisition of new information, serving continuous cognitive shifting, is primarily elaborated through the anterior/vHPC rather than through the posterior/dHPC.

Considering such findings and in connection with previous evidence related to the role of the PFC in altered cognitive flexibility induced by SCZ, the most recent hypothesis of a PFC-HPC “interactive crosstalk” has been highlighted.

### 3.2 Electroencephalography

Electroencephalography (EEG) is known to be a non-invasive method for measuring real-time electrical activity in the superficial layers of the brain, covering an area of approximately 10 cm^2^. This activity discharges synchronously and is derived from a combination of excitatory postsynaptic potentials (EPSPs) and inhibitory postsynaptic potentials (IPSPs). The summation of EPSPs and IPSPs over a selected cortical region with synchronous discharge creates an electrical field, which the EEG then measures. Unlike the fMRI, the EEG offers precise millisecond-level representations of cortical activity, albeit with poor spatial resolution due to its inability to record electrical activity from depths lower than a few millimeters from the scalp. Nevertheless, it remains one of the most effective methods for studying neural communication across various brain regions, especially at the cortical level.

The commonly encountered waveform frequencies in EEG are *α* (8–12 Hz), *β* (15–30 Hz), *γ* (30–120 Hz), Ө (4–12 Hz), and *δ* (less than 4 Hz). Each of the bands is closely associated with a corresponding cognitive and emotional process (Abo-Zahhad et al., 2015). For instance, the γ and Ө bands are associated with attention and memory in the PFC and HPC (Zhang et al., 2022; Patrono et al., 2023, 2025; Bai et al., 2024), whereas the α band is linked to relaxation and sensory processing (Abo-Zahhad et al., 2015). The same way that the EEG studies have highlighted the interaction between wave bands and cognition/emotion, the studies also provided some interesting insights regarding changes in band oscillations synchrony in the PFC and the HPC, which in turn showcased altered cognitive performance (Weiss and Rappelsberger, 2000; Weiss et al., 2000; Summerfield and Mangels, 2005).

In the study by Donati et al. (2024), a combined approach of EEG and Transcranial Magnetic Stimulation (TMS-EEG) was used to observe oscillatory activity in the dlPFC of early-course SCZ (EC-SCZ) patients. The study found that these patients demonstrated significantly lower natural frequencies in the dlPFC compared to the control group, which suggests that the prefrontal neurons of these individuals are firing at an abnormally slow rate. As previously stated, there is a connection between the oscillatory bands and emotions and cognition. In this research, oscillatory activity in the PFC, within specific frequency bands, is known to support higher-order cognitive functions, including attention and decision-making. Therefore, a disruption in these specific oscillatory synchronies may directly reflect a similar breakdown in executive processes. It is also interesting that EC-SCZ patients showed poorer working memory performance, which was negatively correlated with increased *β*-band power in the dlPFC, revealing a pattern directly opposite to what is typically observed in healthy cognitive processes (Barone and Rossiter, 2021). These results expand on the idea that abnormal PFC oscillations interfere with communication across brain regions, contributing to the cognitive and motivational deficits observed in early-stage SCZ. Lastly, the EC-SCZ patients who had lower dlPFC frequencies also demonstrated more severe negative symptoms (e.g., apathy or social withdrawal), which may imply that reduced PFC synchrony is an early biomarker of the disorder.

Building on the previous evidence, another study explored the use of High-Definition Transcranial Direct Current Stimulation (HD-tDCS) (Parlikar et al., 2021). This method delivers very weak electrical currents to both cortical and deeper brain regions. They delivered mild electrical current through electrodes attached to the scalp of patients to modulate neural activity in the dlPFC. Fifty-nine individuals with SCZ were randomly allocated to receive either the real or a fake HD-tDCS, specifically targeting their left dlPFC. The primary objective of this research was to determine whether stimulating this region could alleviate negative symptoms, such as anhedonia and demotivation, as measured by the Positive and Negative Syndrome Scale (PANSS). The results showed a significant improvement and mitigation of negative symptoms in the real HD-tDCS group compared to the placebo group. Moreover, negative symptoms remained low in a one-month follow-up examination. Similar to previous studies, Parlikar et al. (2021) observed a decrease in *β*-band oscillatory activity. The β-band has also been seen to contribute to functional connectivity within the DMN framework (Whitton et al., 2018; Wang et al., 2025), thus suggesting that modulating PFC activity may help restore functional connectivity in several brain regions of SCZ patients.

Another interesting study focused on the DMN connectivity framework, comparing multiple psychiatric disorders using graph theory analysis— a mathematical approach to studying complex brain networks — and EEG (Choi et al., 2021). While reviewing the brain scans, the researchers noticed that SCZ patients exhibited significantly higher *θ*-band hyper-clustering in the Fronto-Temporo-Parietal regions. This suggests significant disconnections or inefficiencies in network communication, which correlated with cognitive impairments. However, an interesting similarity was noted between SCZ and Alzheimer’s disease (AD), as they shared a similar θ-band hyper-clustering (Krukow et al., 2020). On the other hand, disorders with anxiety symptoms (PTSD, panic disorder, MDD) showed hyper-clustering in higher-frequency bands (β and *α*). Furthermore, the study highlighted disrupted θ-band connectivity between the left HPC and the left lingual gyrus, supporting the notion that HPC dysfunction is linked to cognitive decline in SCZ, as HPC θ-waves are crucial for episodic memory and cognitive flexibility. Taken together, these pieces of evidence suggest that differences in brain oscillations or desynchronization of oscillations among different brain regions may be considered biomarkers of SCZ-related cognitive impairment. Therefore, SCZ-related cognitive deficits may originate from disrupted network communication rather than simple regional dysfunction. More importantly, it is demonstrated that PFC-HPC network dysfunction is prevalent in individuals with SCZ.

## 4 Animal models of SCZ

Animal models of psychopathologies play an essential role in basic research, mainly because they allow a better understanding of pathological conditions and aetiopathogenesis that is difficult to reproduce in humans. Moreover, the use of animal models of psychiatric disorders, such as SCZ and other psychosis-related conditions, addresses construct, face, and predictive validity. This approach ensures not only the quality of translation from animal to human but also allows for the investigation of aetiopathological phenomena, including genetic factors and bio-physiological brain patterns.

### 4.1 Neurodevelopmental and genetic animal models of SCZ

Animal models that mimic early-life conditions and genetic disruptions are crucial in demonstrating the developmental origins of SCZ, particularly concerning dysfunctions within PFC-HPC connectivity. Neurodevelopmental risk factors, such as immune activation, excitotoxic lesion models, or disrupted gene expression during critical periods, can alter the maturation of interneurons and neural circuits, resulting in long-term cognitive and network-level abnormalities (Lipska and Weinberger, 2000; Tseng et al., 2009; O'Donnell, 2012; Bitzenhofer et al., 2021). In this section, four established models are prominently discussed: maternal immune activation (MIA), deletion of voltage-gated potassium channels type 3 (Kv3), Ca2+/calmodulin-dependent protein kinase II-α (CaMKIIα) knockout, and the neonatal ventral hippocampus lesions (NVHL) rodent model. Such models highlight the theory that early disturbances impair oscillatory coordination between the PFC and HPC, and may highlight the “PFC–HPC loop” not only as a component of SCZ pathophysiology but also as a core etiological factor.

In the MIA model, an SCZ-like phenotype may be observed in the offspring of pregnant rodents that were exposed to immune challenges, such as the Polyinosinic: Polycytidylic Acid (Poly I: C) - a synthetic compound which mimics double-stranded RNA and acts as an immunostimulant (Meyer and Feldon, 2012, for review). A recent study used the MIA model to investigate the role of temporal organization of memory in the HPC by observing HPC-related *θ*-phase precession (coordinating HPC cell assemblies such that different cells fire in sequence within a single θ cycle) and θ sequences (representing time-compressed trajectories through space) (Speers et al., 2021). Despite single-cell precession remaining unimpaired, MIA rodents displayed increased inconsistency throughout the initiation phase of precession, thus resulting in disorganized θ-phase precession sequences. Because phase precession significantly contributes to HPC-PFC synchrony, these findings suggest that early immune challenges disrupt a neural timing mechanism necessary for coherent PFC-HPC communication, which may be a potential origin of SCZ-related deficits in working memory and narrative thought (Wang et al., 2024).

Genetic models targeting ion channel function further support the theory regarding dysfunctions in the PFC-HPC connection. The Kv3 family of voltage-gated potassium channels, particularly Kv3.1 and Kv3.3, plays a crucial role in high-frequency firing in parvalbumin-expressing interneurons (PV), which are essential for generating *γ* oscillations. In a recent study, Condon (2024) utilized Kv3.1 and Kv3.3 knockout mice to investigate the role of these channels in contributing to overall network synchrony. The recordings from awake, head-fixed mice showed an increasing shift in the oscillatory balance, marked by reduced low-frequency synchrony and significantly high-frequency activity. This result suggested a disruption in the excitatory-inhibitory balance within the PFC-HPC circuit. Furthermore, this supports the expanding idea that SCZ is a disorder characterized by circuit-level dysconnectivity.

Another model focused on the heterozygous knockout of a specific protein, CaMKIIα, which is important for synaptic plasticity. Interestingly, mice with this particular alteration exhibited long-term memory impairments, reduced overall activity, increased θ power in the HPC, and some spontaneous spike–wave bursts (Featherstone et al., 2022). Typically, higher θ activity is associated with cognitive activities; however, in this case, the elevated levels most likely reflect abnormal communication between brain regions rather than typical functional cooperation. Furthermore, the mice exhibited changes in their event-related potentials (ERPs) and delayed neural response patterns, indicating timing and sensory processing deficits —symptoms commonly observed in SCZ patients.

Moreover, NVHL in rats produces an animal model commonly used to document the neuro-developmental aspect of SCZ (Lipska and Weinberger, 2000; Tseng et al., 2009). It consists of causing vHPC damage at the end of the first week of life. Lipska and coll. Induced excitotoxic lesions activating NMDA receptors in neonatal stages to investigate the role of developmental stages of SCZ-like inabilities later measured in adulthood. With the help of MRI, the damage can be visualized 2 weeks later. Even if no such lesions exist in SCZ patients, they seem to modify brain development in a similar way, which is now considered crucial for the construct validity of the model (O'Donnell, 2012).

Finally, the animal models presented here show how early-life or genetic disruptions impair the oscillatory integrity of PFC-HPC circuits. Whether through immune activation, channel disruptions, or altered synaptic signaling, these mechanisms underscore the common notion that disorganized rhythmic communication contributes to cognitive dysfunction in SCZ.

### 4.2 Structural and functional alterations in the HPC and PFC in animal models of SCZ

Similar to *in vivo* research with human participants, several studies using rodent models discuss how structural and synaptic abnormalities in the PFC and HPC contribute to SCZ symptoms and characteristics. Therefore, studies using rodents further support and mirror studies with human participants, showing various kinds of PFC-HPC maladaptive activities like the hyperactivity in HPC circuits, synaptic pruning deficits, and imbalances in excitatory and inhibitory neurotransmission (Pafundo et al., 2021, for review), further suggesting that aberrant PFC-HPC communication leads to cognitive dysfunction.

One of the key models in this debate is the overexpression of the immune-related gene complement C4, which is associated with an increased risk of SCZ. Druart et al. (2021) found that elevated C4 expression in the PFC reduced glutamatergic input, caused fewer dendritic spines, and impaired GABAergic transmission, specifically in PV interneurons. GABA is the primary inhibitory neurotransmitter, released specifically at GABAergic synapses, functioning as a mediator in fast-acting phasic inhibition (Koh et al., 2023). Additionally, PV interneurons are calcium-binding cells (Permyakov and Uversky, 2022) that play a crucial role in regulating the balance between excitation and inhibition in the brain, which is essential for smooth communication and proper functioning (Godoy et al., 2022). Intriguingly, Druart et al. (2021) noted that a disbalance between the excitation and inhibition was associated with working memory deficits, suggesting again a circuit-level disruption relevant to SCZ. However, this model highlights explicitly how immune-mediated synaptic pruning can affect network connectivity and impair cognitive functions.

Wegrzyn et al. (2022) analyzed the results and evidence across multiple SCZ animal models and, similarly to Druart, concluded that HPC overactivity is consistently observed across both early and chronic stages of the disorder. HPC hyperactivity is typically associated with structural changes, including reduced interneuron density and imbalances in glutamate and/or GABA, as observed in previously mentioned animal and human studies. Therefore, HPC overactivity may contribute to the disruption of PFC-HPC communication, as it destabilizes oscillatory synchrony within the PFC. This further impairs cognitive processes, such as decision-making, memory encoding, and retrieval. Lastly, Nitta et al. (2021) observed that suppressing the Piccolo protein (a presynaptic cytomatrix protein involved in neurotransmitter release) in the mPFC leads to reduced synaptic proteins and impaired electrophysiological signaling. Mice that had suppressed Piccolo protein showed SCZ-like behaviors (e.g., cognitive deficits and locomotor hyperactivity). Moreover, it was observed that mice exhibiting SCZ-like behaviors had altered dopaminergic and glutamatergic responses in the dorsal striatum, which plays a role in voluntary movements and habits (Goodman and Packard, 2018). The results of this study support the idea that synaptic imbalances, resulting from molecular disruption, can contribute to network-level disconnection and behavioral abnormalities.

These studies examined various models, further supporting the notion that SCZ may not be a disorder of isolated brain regions, but rather a dysfunction of dynamic and interconnected networks. These studies demonstrated how the alterations within the PFC and the HPC disrupt smooth communication, further impairing the symptoms of SCZ.

## 5 The maladaptive PFC-HPC crosstalk in SCZ

The *in vivo* research paved the groundwork for understanding SCZ and its symptoms. Moreover, it enabled a view of specific patterns that lead to further development and reconstruction of the informational framework surrounding the SCZ. Therefore, it is essential to investigate the PFC-HPC functional connectivity through research that specifically examines the interaction between the PFC and the HPC, rather than considering them separately. By focusing on these interactions, a better understanding can be formed regarding how the disrupted communication between them contributes to the cognitive dysfunction in SCZ. Additionally, this approach can help determine whether such a breakdown in connectivity is due to chance or a consequence of the broader cognitive impairments observed in SCZ.

As stated previously, while isolated studies of the PFC and the HPC functioning may reveal how specific dysfunctions in each region contribute to the symptoms of the disorders, this approach is somewhat reductionist, as it overlooks the deeper insights a researcher may gain from exploring their functional connectivity. It may also further clarify if the disrupted communication between the PFC and the HPC drives cognitive dysfunction or if it arises as a consequence of it.

As previously discussed, this interesting interaction is supported by coordinated oscillatory activity, enabling the PFC and HPC to exchange information in real-time (Shing et al., 2022; Ferraris et al., 2021). Sigurdsson and Duvarci (2016) suggested that these regions communicate via indirect anatomical pathways, which provide them with more efficient integration across circuits. Therefore, the central hypothesis is that if HPC-PFC connectivity is crucial for higher-order cognition, then its disruption should be consistently observed across various models of SCZ, driving cognitive dysfunction.

On the other hand, recent research emphasizes that SCZ involves dynamic changes in network interactions (triple network hypothesis) over time, and that a “temporally dynamic” PFC-HPC connectivity may be critical for understanding SCZ-related cognitive dysfunction. Compared to healthy individuals, individuals with SCZ often exhibit reduced, less stable, and more variable dynamic connections between key brain networks, such as DMN, CEN, and SN. These temporal fluctuations in network activity, which are critical for normal cognitive functions, are disrupted in SCZ and can correlate with symptom severity (You et al., 2022). Measuring dynamic functional connectivity provides a more comprehensive picture of brain function than traditional “static” measures, revealing the dynamic fluctuations that are vital for complex cognitive processes. Moreover, understanding these dynamic changes may offer insights into the underlying neural mechanisms of SCZ and help develop more targeted treatments, as some studies suggest antipsychotic treatment can modulate these dynamic PFC-HPC functional connectivity states (You et al., 2022; Wang C. et al., 2021; Wang X. et al., 2021; Wang Y. et al., 2021).

Within this section, a special focus will be placed on the potential function of relay structures, such as the nRE, and oscillatory synchrony, which facilitate coordinated activity across various brain regions (Cassel et al., 2013; Dolleman-van der Weel and Witter, 2020; Patrono et al., 2025). The supported research will demonstrate how the circuit breaks down in SCZ and why the focus should be on functional rather than only anatomical connectivity, considering the role this plays in SCZ and its cognitive impairments.

### 5.1 Individual and cooperative roles of the PFC and the HPC

Although these regions have anatomical differences and are not directly adjacent, the PFC and the HPC still engage in bidirectional communication, typically associated with goal-directed cognitive functions. Some examples of bidirectional communication between these two regions include how the HPC provides contextual and episodic information for the PFC. Through that, the brain can proceed with decision-making based on past experiences. Moreover, the PFC may send goal-relevant signals to the HPC, which in turn allows the brain to proceed with shaping memory retrieval or guide particular behavior (Place et al., 2016). This communication between various brain regions is also referred to as the reciprocal loop or the intention-recollection cycle (Shapiro et al., 2014). This reciprocal loop operates through oscillatory synchrony, as observed in the *θ* and *γ* bands previously mentioned, and is integral to healthy functioning in working memory, spatial navigation, and general cognitive flexibility. However, when this communication breaks down, significant shifts occur in the normally functioning system, such as deficits in memory-guided behavior, impaired decision-making, and cognitive decline. This leads to the hypothesis that an imbalanced excitatory/inhibitory ratio explains the cognitive impairments in SCZ, as previously presented in the sections dedicated to human and animal studies. Based on the description of the various subregions and correlating back to the described studies, it may be deduced that these impairments may not be due to isolated damage with either the PFC or the HPC, but the failure of the general network coordination between various subregions, which are mediated by structures like the nRE and supported by the oscillatory synchrony.

### 5.2 The role of relay structures

A critical point arose after discussing the interdependent and independent functions of the PFC and the HPC. These two brain regions lack direct monosynaptic connections (Vertes et al., 2006; Witter et al., 2000). Therefore, this absence of a direct connection raises the question of how these regions communicate, especially during tasks that require cognitive control. The answer to this question may be found within the relay network structures of the thalamus, where certain regions of the brain act as mediators and synchronizers between areas. In the case of the PFC-HPC loop, the nRE within the thalamic nuclei appears to be the key mediator (Cassel et al., 2013; Dolleman-van der Weel and Witter, 2020; Patrono et al., 2025; Place et al., 2016; Vertes et al., 2006; Witter et al., 2000; Panzer et al., 2024). While structures like these indeed help bridge the anatomical gap between regions like the PFC and the HPC, it is not only the physical pathway that is important in the context of general smooth cooperation and communication. Functional connectivity, which is the synchronized activity between brain areas, is equally important in supporting efficient communication and coordination. Interestingly, functional links like these may exist even without a direct anatomical connection, highlighting the importance of network dynamics in shaping cognition.

### 5.3 The thalamus and the nucleus reuniens: a cognitive relay

The thalamus is predominantly viewed as a relay hub that transmits sensory and motor information to the cerebral cortex; however, it also plays a crucial role in regulating functions such as sleep, alertness, and consciousness (Fang et al., 2025). Within the thalamic structures, there are also specific subregions, such as the mediodorsal thalamus and the nRE, which are theorized to support executive processes, memory, and attention by facilitating coordination between the PFC and HPC (Cassel et al., 2013; Dolleman-van der Weel and Witter, 2020). Moreover, it was found that reduced connectivity between the mediodorsal thalamus and the dlPFC was associated with impairments in working memory and executive function in SCZ individuals (Shin and Jadhav, 2016; Giraldo-Chica et al., 2018). These alterations often appear early on in the course of the disorder. Yet, they also tend to persist as the disease progresses, which further supports the theory that, at least in part, SCZ may stem from a broader breakdown in brain network communication, rather than just from isolated regional abnormalities (Haber et al., 2021).

**Figure 1:**
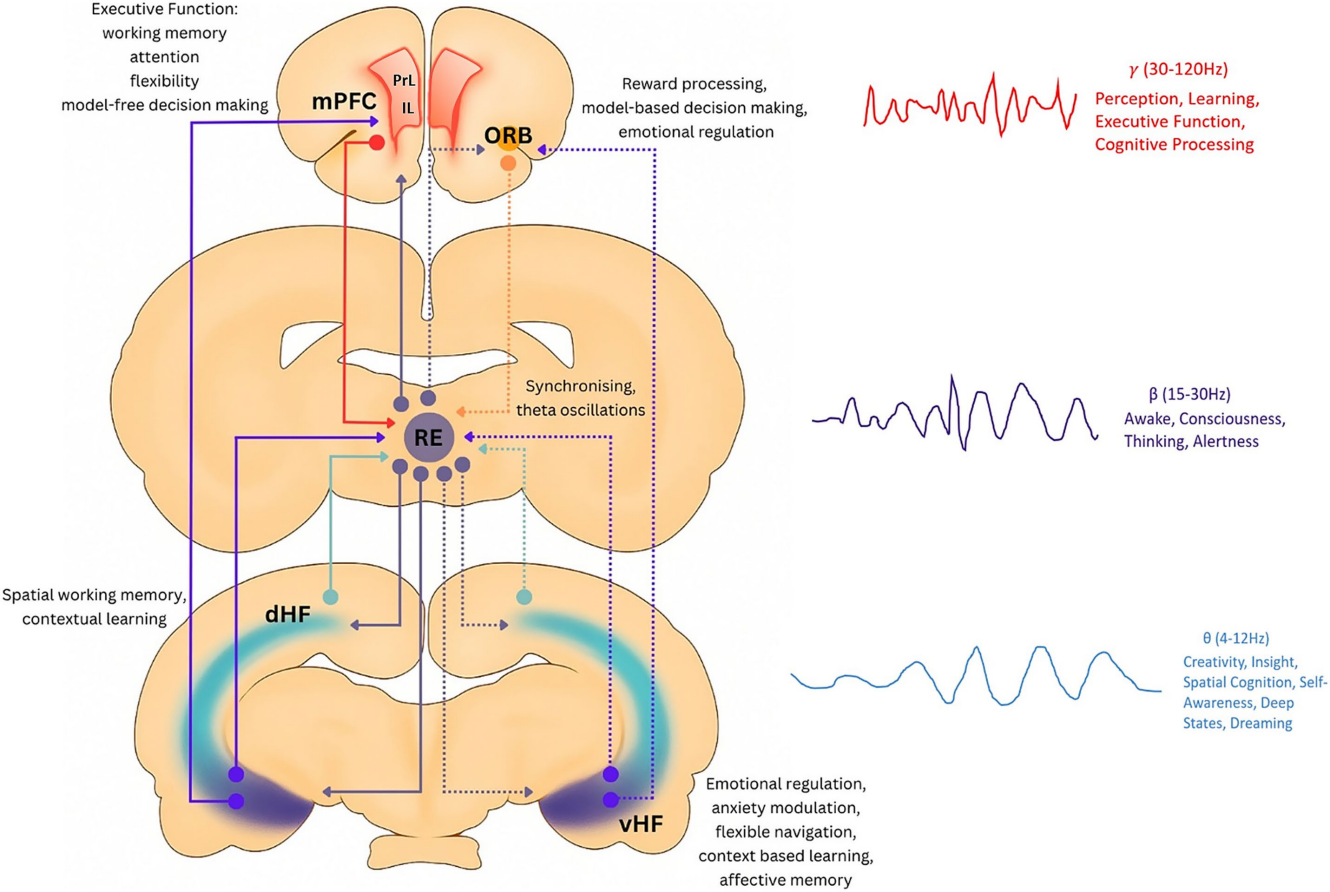
Representative circuit formed by prefrontal-hippocampal (ventral and dorsal) connections with the nucleus reuniens. The neuronal terminals from the mPFC form a direct synaptic contact (purple lines) on the dendrites of the vHPC, which further project onto the nRE. These connections are recruited during executive functions such as spatial working memory, contextual learning, and model-free decision-making (Padilla-Coreano et al., 2016). On the other hand, indirect mPFC (red line)-dHPC (light blue line) pathways through the nRE serve attention, flexibility, and reward processing through model-based decision-making (Mugan et al., 2024). Moreover, the nRE-ORB (dotted purple line) pathway is involved in attentional flexibility (Rojas et al., 2024). Furthermore, the nRE supports oscillatory coherence within the PFC-HPC communication, specifically in the *θ* (4-12 Hz) and *β* (15-30 Hz) ranges (for the vHPC-nRE connection, indicated by the dotted purple line; and for the dHPC-nRE connection, indicated by the dotted light blue line) and the *γ* (30-120 Hz) and β ranges (for the mPFC-nRE connection, indicated by the dotted dark purple line; and for the ORB-nRE connection, indicated by the dotted orange line). In this context, temporal fluctuations in the functional relationships between different brain regions, namely dynamic connectivity patterns, provide an accurate picture of brain network activity. Given that the information flow in the brain is causally organized in time and frequency-based wave oscillations, dynamic connectivity models could be more informative in terms of understanding brain function and investigating brain-behavior associations. These patterns, analyzed using techniques like dynamic functional connectivity (DFC), reveal how brain regions reconfigure their interactions over short time scales in response to task demands, cognitive states, or disorders. DFC often uses fMRI data to track these changes by looking at time courses of neural activity in different brain areas (Jalilianhasanpour et al., 2021). Thus, it is hypothesizable that temporal variability in PFC-nRE-HPC connectivity will correlate with pharmacological antipsychotic response in SCZ subjects. This is supported particularly during tasks such as engagement with working memory, spatial navigation, or rule switching (modified from Vertes et al., 2022).

Within the thalamus, a subregion coordinates PFC-HPC communication at the ventral midline, the nRE. Briefly, the nRE functions as an informational relay, receiving informational projections from the PFC and sending reciprocal outputs to the CA1 of the dHPC, thereby forming a bidirectional circuit among the brain regions, with both anatomical and functional connections observed in animal models of psychiatric disorders (Vertes et al., 2006; Cassel et al., 2013; Dolleman-van der Weel and Witter, 2020; de Mooij-van Malsen et al., 2023). However, it is essential to note that recent evidence suggests the HPC may not be a major target of the nRE projections, as previously assumed. A 2025 study using advanced circuit-tracing methods found “no evidence that pyramidal cells in CA1 receive input from NRe, with midline thalamic input to hippocampus proper appearing selective for GABAergic interneurons” (Andrianova et al., 2025). In addition to this, studies from our group have shown that optogenetic activation of PV + interneurons rescues SCZ-like cognitive impairments induced pharmacologically by selective inhibitors of Glutamergic NMDAR (Patrono et al., 2023, 2025). Moreover, it has been observed that the nRE in rodents does not contain GABAergic terminals, but only Calretinin/Calbindin (CR/CB+) neurons (Dolleman-van der Weel et al., 2019; Viena et al., 2021; Vertes et al., 2022). However, another study in 2022 (Joyce et al., 2022) found GABAergic terminals in the nRE in macaques. Nonetheless, because both PV + and CR/CB + are calcium-binding proteins, the hypothesis is that specific calcium channels (CaV3.2), which are low-voltage-activated channels, are vital for neuronal excitability, sensory processing, sleep, and pain, and are involved in hippocampal-nRE-prefrontal communication (Lory et al., 2020). Figure 1 illustrates a representative circuit formed by prefrontal-hippocampal (ventral and dorsal) connections with the nucleus reuniens (nRE). The neuronal terminals from the PFC form a direct synaptic contact on the dendrites of the vHPC, which in turn project their terminals into the nRE. However, indirect connections are formed between the mPFC and the dHPC via the nRE. Furthermore, the nRE supports oscillatory coherence within the PFC-HPC communication, specifically in the *θ* and *β* ranges (for the vHPC-to-nRE connection) and the *γ* and β ranges (for the mPFC-to-nRE connection). This support is particularly prominent during tasks such as engagement with working memory, spatial navigation, or rule switching (de Mooij-van Malsen et al., 2023). The maladaptive communication, specifically in the functioning of the nRE, occurs if there are lesions or specific disconnections of the nRE itself from the general circuit. This alteration may lead to behavioral flexibility issues and impaired decision-making, particularly when it is associated with memory (Ferraris et al., 2021; Patrono et al., 2025).

Additionally, research indicates that dopaminergic modulation, specifically the D4 receptor, further supports the functions of the nRE in enhancing attention or potentially compensating for a disrupted top-down control (Kuang et al., 2023). The dopaminergic modulation aids with the *δ*-band coherence between the three regions: the PFC, the HPC, and the nRE. Finally, it has been suggested that the nRE mediates communication through oscillatory synchrony with the θ, β, and γ bands across the mPFC, nRE, and CA1 of the vHPC (de Mooij-van Malsen et al., 2023). This proposes an alternative illustration of the model, along with more precise and distinct subregions. Moreover, these references enable the audience to form ideas about the potential functional connectivity between the well-known regions involved in SCZ and the lesser-known thalamic subregion.

### 5.4 Functional connectivity of the PFC-nRE-HPC

Functional connectivity is theorized to arise predominantly through oscillatory coherences and relay circuits, such as the nRE and, in part, the basal ganglia (Shing et al., 2022; Ruggiero et al., 2021). As already mentioned, each oscillatory rhythm has its corresponding role, which plays an essential part in one’s functioning, especially within the PFC and HPC regions. All subsequent examples discussed correspond to the PFC-HPC connectivity. For instance, the 𝛾 synchrony plays a role in the temporal coordination of memory encoding and retrieval (Sigurdsson and Duvarci, 2016; Shin and Jadhav, 2016). Additionally, the 𝛾 synchrony aids top-down control during navigation flexibility tasks (Patrono et al., 2025). Lastly, the two bands that are increasingly recognized as mediators of long-range communication across relay nodes (like the thalamus and basal ganglia) are the δ and β rhythms (Kuang et al., 2023; Jayachandran et al., 2023).

The main points highlighted in this paper are that: (a) functional connectivity in the brain is needed for complex, higher-order cognitive abilities; (b) functional connectivity among brain regions is predominantly determined by oscillatory activities of specific areas that must be coherent and coordinated, possibly supervised by relay substructures, such as the thalamic nRE. However, in our view, two questions remain to be fully elucidated: (1) whether functional connectivity is required for higher-order cognitive abilities, and if such abilities are generally considered to be either domain-general or domain-specific, is functional connectivity domain-general or domain-specific? (2) If the oscillatory activities of specific areas must be coherent and coordinated to support functional connectivity, what is the possible physiological “common denominator” that allows such oscillatory coherence and coordination, especially in the nRE?

To answer these questions, two main frames should be addressed: (a) the theoretical frame of domain-general/domain-specific functional connectivity; and (b) the neurophysiological frame of the “common denominator” of oscillatory coherence among distinct brain structures.

#### 5.4.1 The theoretical frame of functional connectivity

As previously described, the concept of domain-specific processes refers to cognitive skills related to specific types of information, such as language, social understanding, and spatial awareness, which are associated with corresponding brain regions.

On the other hand, domain-general processes reflect the ability to coordinate across various cognitive domains, supporting functions such as working memory, attention, and cognitive flexibility. The domain-general capacities and functionings are also typically described as an “orchestrated” working, as they combine and integrate different systems and approaches for unified thinking and behavior (Li et al., 2014). However, regarding functional connectivity, which refers to the temporal correlation between different brain regions, it can be either domain-specific (strongly associated with a particular cognitive function) or domain-general (involved in multiple cognitive functions). Domain-specific connectivity highlights the specialization of brain regions for specific tasks. For example, the fusiform face area (FFA) and the parahippocampal place area (PPA) show strong domain-specific connectivity, indicating their specialized role in face and scene perception, respectively (Kamps et al., 2020).

In contrast, domain-general connectivity suggests a more general, adaptable network that supports various cognitive processes. For example, the frontoparietal network, also known as the multiple-demand network, exhibits domain-general connectivity, indicating its role in various cognitive control tasks (Bulut, 2023). Nonetheless, it’s important to note that domain-specific and domain-general connectivity are not mutually exclusive. Brain regions can be part of both domain-specific and domain-general networks. For example, working memory is widely involved in all domains of cognition and is thus considered a domain-general ability. However, it has recently been reviewed that the domain generality of working memory varies substantially in terms of computations (*in silico* models of neural networks), neural correlates, and applications (training models for rehabilitation programs). This indicates that, computationally, working memory is largely domain-general. In terms of neural correlates, it contains both domain-general and domain-specific elements. Finally, in terms of application, it is domain-specific mainly (Nozari and Martin, 2024). Understanding the interplay between domain-specific and domain-general connectivity is crucial for understanding how the brain functions and how different higher-order cognitive processes are organized. This knowledge can also be applied to understanding and treating neurological and psychiatric disorders, as well as understanding the progression of executive function organization across developmental stages (Schäfer et al., 2024).

#### 5.4.2 The neurophysiological frame of functional connectivity

As previously stated, the PFC-HPC functional connectivity arises from oscillatory coherences and relay structures, like the nRE. Specific waves of oscillations in particular brain structures coordinate specific cognitive functions: θ and 𝛾 synchronous waves in the HPC play a role in the temporal coordination of memory encoding and retrieval (Sigurdsson and Duvarci, 2016; Shin and Jadhav, 2016); 𝛾 synchrony in the PFC aids the top-down control during flexibility tasks (Patrono et al., 2023, 2025); *δ* and *β* rhythms mediate of long-range communication across relay nodes (Kuang et al., 2023; Jayachandran et al., 2023).

Furthermore, it has recently been hypothesized that the oscillatory coordination of such waves might be governed by an excitatory/inhibitory (E/I) balance in the synaptic activity of a neuronal network (i.e., the prefrontal-hippocampal circuit for executive functions) (Szczurowska et al., 2017; Ferguson and Gao, 2018). The E/I plays a vital role in the brain’s normal functioning, controlling, for example, the regular spike rate. It is represented by the inhibitory GABAergic and excitatory glutamatergic N-Methyl-D-Aspartate Receptors (NMDAR), which balance transmission from molecular to systemic levels (Szczurowska et al., 2017). This delicate balance is perturbed in SCZ, autism, and OCD. In particular, the firing rate of PV neurons decreases, while the firing rate of excitatory neurons increases, resulting in paradoxical hyperexcitation due to disinhibition (Homayoun and Moghaddam, 2007). Thus, PV plays a crucial role in regulating local excitatory activity through robust inhibitory control and is also essential in generating 𝛾 oscillations. Recently, we observed that optogenetic stimulation of PV at *γ*-like frequencies (50 Hz) or *θ*-like frequencies (10 Hz) in the mPFC or vHPC, respectively, rescued MK801-induced alterations in flexibility (Patrono et al., 2023, 2025). This suggests a role for PV in specific brain regions (domain-specific, including the mPFC and vHPC) during higher-order cognitive functions. It is crucial to note that although flexibility can be restored using a domain-specific approach (mPFC or vHPC PV local optogenetic stimulation), healthy flexibility may also be regulated through a more extensive neural network under a more domain-general condition. Therefore, the nRE might be pivotal in balancing the local mPFC-vHPC PV activity under more domain-specific conditions.

A central question arising from recent investigations is whether it is possible to link the mPFC, vHPC, and nRE via the GABAergic PV (Vertes et al., 2006; Cassel et al., 2013, 2021). However, counterintuitively, unlike other thalamic nuclei, the nRE lacks PV interneuron terminals (Graham et al., 2021). On the other hand, nRE is primarily composed of calretinin-positive (CR+) neurons, which have terminals that extend to the ventral CA1 and subiculum of vHPC (Dolleman-van der Weel and Witter, 2020). CR + neurons express calcium-binding proteins, as do the PV interneurons. However, CR + and PV form isolated subpopulations that do not overlap and have different morphological and electrophysiological characteristics. PV appears mostly on non-pyramidal neurons (GABAergic basket cells). In contrast, CR + appears mostly on projection neurons between layers 2/3 and 5/6, thus suggesting that PV and CR + form an integral network component that controls the thalamic outputs to the vHPC and mPFC.

This suggests that PV and CR + form an integral network component that controls the thalamic outputs to the vHPC and mPFC, utilizing a “common denominator” that underlies the mPFC-nRE-vHPC communication. Recent evidence has demonstrated phase-locking activity between the nRE and vHPC during sleep (Basha et al., 2025), as well as synchronized θ-band oscillations between the nRE and vHPC during spatial working memory (Hallock et al., 2016). Moreover, both CR + and PV express specific low-threshold T-type Ca2 + channels (CaV3.2), which in the nRE generate β-like frequency-firing activity correlated with memory consolidation and retrieval, as well as contextual novelty (Jayachandran et al., 2023; Walsh et al., 2025). Ca2 + is an essential second messenger, and its entry can depolarize the plasma membrane, thereby activating other voltage-gated ion channels. This property is significant for neuronal CaV 3.2 channels, which can generate low-threshold spikes that lead to burst firing and oscillatory behavior (Perez-Reyes, 2003). Finally, mounting evidence is converging on the role of calcium signalling in the thalamic nuclei in “orchestrating” (coordinated coherence) the various oscillations coming from different brain structures, which subserves the domain-general functional connectivity needed for higher-order cognitive abilities. Thus, it is hypothesized that thalamic calcium-signaling through the CaV3.2 channels might be the physiological “common denominator” of the PFC-nRE-HPC functional connectivity.

## 6 *In silico* evidence: computational models

As we have shown here, recent advances in brain imaging and other experimental techniques have provided strong evidence for anatomical (structural) changes and functional connectivity disturbances in SCZ and other disorders affecting executive functions. They also reveal the link between specific psychological symptoms and their neural correlates (the psychophysical problem).

While *in vivo* studies provide valuable and solid insights into the PFC-HPC connectivity observations, they are not immune to certain limitations. *In vivo* studies are prone to isolating specific mechanisms (Fröhlich and Salar-Behzadi, 2014). This is understandable, as it may not be as efficient to scan or investigate minor intricacies that might not be present. Moreover, subjective differences across SCZ patients are not taken into account. Therefore, various covariates may exist that can lead to imprecise results. In this case, researchers proposed using computational modeling (Battaglia and Brovelli, 2020) to envision regional or oscillatory changes across brain regions, specifically in the context of SCZ. These *in silico* models can utilize pathological or normal conditions to observe changes in SCZ-like simulated brain structures, enabling researchers to investigate complex scenarios in a significantly more controlled and precise manner (Battaglia and Brovelli, 2020). It is essential to note that these models are not proposed as a replacement for in vivo models. Generally, it is known that *in silico* models of SCZ require numerous assumptions about parameter values that are often not empirically constrained. Building complex computational models to simulate SCZ requires selecting numerous parameter values that usually lack direct biological evidence, highlighting the inherent limitations and challenges in developing computationally robust models for this complex neurological disorder (Kurtin et al., 2023). Instead, they serve as a supporting tool to complement existing in vivo results. Thus, *in silico* models are not only an additional validation method but also provide a platform for hypothesis generation and the controlled exploration of complex mechanisms that are difficult to isolate in vivo. Validating computational biological models involves comparing model outputs to experimental data using statistical metrics and evaluating for emergent behavior and biological plausibility. This requires careful model calibration through techniques like parameter estimation and data assimilation to align model parameters with real-world observations. Validation also includes ensuring the model is internally consistent, can be reproduced independently, and has been reviewed by domain experts who are not model developers (Fitzpatrick and Stefan, 2022; Tatka et al., 2023).

One computational study developed an HPC CA3 model (Sherif et al., 2020). This model simulated disruption in the interaction between the NMDA/GABA receptors and the hyperpolarization-activated current (*I*h). The computational simulations showed that the most appropriate state for smooth communication with well-synchronized 𝛾 waves was achieved when these receptors and currents were adequately balanced. The research further states that the activity of this simulation follows inverted U-shaped functions. Here, the inverted U-shaped function refers to cognitive impairments that can be observed under both hypermodulation and hypomodulation of the neuronal firing activity. Authors demonstrate that both excessive and insufficient modulation of NMDA, GABA, or Ih conductances result in reduced information transfer, following an inverted U-shaped relationship. In this framework, cognitive impairments emerge when neuronal activity is either too disorganized or too rigidly synchronized, disrupting effective communication across the network. Interestingly, some studies directly mirror this exact situation in real-life cases. The EEG findings in SCZ patients indicate that both excessive and reduced synchrony are associated with significantly worse outcomes in cognitive functions (Donati et al., 2024; Gaebler et al., 2023; Gao et al., 2021; Yeh et al., 2024). Concerning the methodologies to validate the criteria used in this model, it would be essential to test both excessive and insufficient modulation of NMDA, GABA, or Ih conductances in clinical and preclinical models. For instance, in SCZ animal models, the synchronization of wave oscillations can be measured using phase-amplitude coupling (PAC) analysis. This analysis quantifies the relationship where the phase of a low-frequency brain oscillation modulates the amplitude of a high-frequency oscillation, indicating neural coordination and information integration (Maheshwari et al., 2017).

Another computational model simulated a pattern separation in the dentate gyrus (DG) of the HPC, specifically reflecting its modulated functioning in SCZ patients (Modhej et al., 2022). This model highlighted the intricacies and sensitivities of such regions, as even the most minor simulated changes in inhibition or excitation disrupted the normal functioning of this region, leading to SCZ-like similarities. This model demonstrated how specific regions influence overall cognitive outcomes and highlighted that these circuits are remarkably vulnerable, potentially underlying higher-order cognitive impairments. To validate this model, it would be interesting to carry out clinical studies on SCZ subjects using fMRI data to track changes by looking at time courses of neural activity in different brain areas (dynamic functional connectivity, DFC), which would help reveal how brain regions reconfigure their interactions over short time scales in response to altered cognitive states.

Another study employed a model of a rodent brain to simulate the chemogenetic inactivation of the nRE (Rojas et al., 2024). They separated the nRE from the other regions, specifically the ORB, simulating a functional disconnection of the areas. Upon disconnection, significant impairments in behavioral flexibility, commonly observed in patients with symptoms similar to SCZ, were noted. The results again mirror real-life examples, which also state that the nRE is an essential region for smooth interregional communication (de Mooij-van Malsen et al., 2023). Therefore, one possible way to validate these results would be to apply complex, multimodal approaches, at least in animal research, where a potential combination of recent techniques and methodologies for the activation of specific actuators, such as synthetic receptors (Designer Receptors Exclusively Activated by Designer Drugs, DREADDS) or photosensitive proteins (channelrhodopsin-2, ChR2), and the use of recording systems, such as single-unit electrophysiology or Local Field Potentials recordings may highlight the specific functionalities of the particular brain regions in specific cognitive abilities. A combination of “activation and recording” methodologies has been recently employed through the implementation of new tools, such as optrodes. These devices combine an optical fiber or micro-LED with microelectrodes, allowing researchers to simultaneously deliver light for optogenetic stimulation and record neural electrical activity. This powerful tool enables precise, cell-type-specific manipulation and simultaneous recording of neuronal circuit activity with high temporal resolution, providing insights into neural circuit function *in vivo*.

In recent years, the “cortical attractor neuronal networks” model has provided a unifying theory that explains the relationship between psychopathology and various levels of brain changes. Notably, it suggests that psychopathology, along with the associated brain changes, can be understood through the lens of how neuronal networks become either unstable or overly stable, thereby affecting cognitive functions such as memory and attention. This model proposes that these networks, composed of interconnected neurons, can settle into specific stable states (attractors) that are crucial for various cognitive processes. Additionally, it suggests that disturbances in these network dynamics can lead to executive dysfunctions, such as those affecting memory, attention, and decision-making (Adams et al., 2018). Such networks are found at three levels of neuronal organization: the micro-scale level (neurons and neuronal networks), meso-scale level (cortical and subcortical regions), and macro-scale level (individuals in social communities) (Looijestijn et al., 2015). There are two main attractor states: (A) a resting state toward which the network tends to flip by default (low energy). This state can represent either the developmentally pre-wired connections (e.g., long white-matter tracts) or the pattern that has been stored in the network by plasticity (learning). When enough energy (e.g., action potentials, sensory inputs) is applied, the network can move to (B) a higher-energy stable attractor state. This situation can be depicted by energy landscapes, which show the basins of attraction as valleys and the attractor states as fixed points within the valleys. When low energy is applied, the network settles into lower-energy attractor states. When more energy is utilized in the form of dendritic input, networks transition from low-energy attractor states to robust, high-energy attractor states, resulting in the subsequent formation of intense percepts (Loh and Deco, 2005).

At the meso scale level (cortical and subcortical regions), the “A-to-B” attractor state condition is developed over time by timely, coherent, and coordinated oscillatory activity of brain rhythms. In pathological conditions, the “A-to-B” attractor state condition does not present any timely, coherent, and coordinated oscillatory activity of brain rhythms. It has been suggested that altered time-scaled attractors might explain the lack of coordination between domain-specific brain regions (Brennan and Proekt, 2023; Taylor et al., 2024). In other words, in psychotic conditions, it is hypothesized that altered executive functions, particularly decision-making, working memory, and flexibility, may result from the desynchronized use of prospective (PFC) and retrospective (HPC) memories within the neural network formed in the thalamic nRE. Very recently, computational network generative modeling has been implemented, such as the recurrent Hopfield neural network (RHNN), in which learning rules (based on the Hebbian plasticity) are identified and algorithmically implemented to produce synthetic network architectures with the same properties, i.e., summary statistics and topology, of the collected data, based on the Hebbian learning principle. Once trained, a generative model can predict unobserved and out-of-sample data. Moreover, an RHNN can reveal the mechanisms that guide the formation of a system and its hierarchical structure (“A-to-B” attractor state condition). To identify a generative model, a set of neural models of communication must be trained, for example, by leveraging available knowledge on structural or functional connectivity or the dynamics of activity. The RHNN is a powerful computational method that can help elucidate the time-dependent synchronous activity of specific brain rhythms, thereby modeling the functional connectivity in the PFC-nRE-HPC circuit of SCZ.

Based on the evidence presented here, it is clear that *in silico* approaches directly mirror in vivo observations, further validating the observed phenomena, particularly in the case of PFC-HPC functional connectivity in SCZ. Furthermore, *in silico* approaches enable researchers to identify and modulate causal mechanisms. They can interact with the model in real-time, allowing for the investigation of both regular and pathological aspects without time or significant manual restrictions. Due to their ability to manipulate simulations in intricate ways, some research suggests that these models may be a key tool for the potential future improvement of predictable, treatment-sensitive biomarkers (Onwordi et al., 2023). Conclusively, it was also suggested that *in silico* modeling approaches may be highly useful for exploring multi-target pharmacotherapy, meaning that simulations like these may provide an integral tool for testing how various pharmacological treatments affect brain functions and, later, help guide the development of alternative therapeutic approaches for disorders like SCZ (Sherif et al., 2020).

## 7 Generated hypotheses and testable predictions

The present study reviewed recent research from in vivo (animal models and human studies) and *in silico* studies (examining functional connectivity). The aim was to stimulate discussions among computational and translational research fields, with further planning for new experimental methodologies. To this, here we present three testable hypotheses generated by considering a few studies presented here.

### 7.1 Hypothesis 1: CaV3.2 channels in the nucleus reuniens

As previously described, both CR + and PV express specific low-threshold T-type Ca2 + channels (CaV3.2), which in the nRE generate *β*-like frequency-firing activity correlated with memory consolidation, retrieval, and contextual novelty (Jayachandran et al., 2023; Walsh et al., 2025). Thus, it is hypothesized that thalamic calcium-signaling through the CaV3.2 channels might be the physiological “common denominator” of the PFC-nRE-HPC functional connectivity. To test this hypothesis, selective chemogenetic modulation of CaV3.2 channels may restore cognitive flexibility in SCZ rodent models. To confirm such selective modulation, electrophysiological measurements of signal coherence and phase-locking activity of the different oscillations should be considered. These measurements quantify the synchronized timing between different oscillations and brain regions, revealing how information is integrated and transmitted across the brain.

### 7.2 Hypothesis 2: domain-general vs. domain-specific PV dysfunction explains cognitive heterogeneity

As previously demonstrated, recent evidence has shown that the optogenetic activation of GABAergic PV interneurons at specific frequencies, ranging from *γ*-like to *θ*-like, plays a pivotal role in rescuing cognitive flexibility, specifically in the forms of attentional set-shifting (Patrono et al., 2023) and navigational set-shifting (Patrono et al., 2025). This suggests that domain-specific dysfunction of the PV impacts its domain-general function. Thus, it is hypothesizable that such a domain-specific dysfunction/domain-general function unbalanced ratio might explain cognitive heterogeneity seen in SCZ. To test this hypothesis, combined optoelectrophysiology methodologies should evaluate signal coherence and phase-locking activity of the different oscillations in several animal models of SCZ. By analyzing the synchronized firing of neurons and the phase relationships between different brain oscillations from these recordings, researchers can assess how altered neural communication contributes to symptoms of SCZ.

### 7.3 Hypothesis 3: dynamic connectivity patterns predict treatment response better than static measures

Dynamic connectivity patterns refer to the temporal fluctuations in the functional relationships between different brain regions, providing an accurate picture of brain network activity (Bolton et al., 2023). Given that the information flow in the brain is causally organized in time, dynamic connectivity models could be more informative in terms of understanding brain function and investigating brain-behavior associations. These patterns, analyzed using techniques like dynamic functional connectivity (DFC), reveal how brain regions reconfigure their interactions over short time scales in response to task demands, cognitive states, or disorders. DFC often uses fMRI data to track these changes by looking at time courses of neural activity in different brain areas (Jalilianhasanpour et al., 2021).

Thus, it is hypothesizable that temporal variability in PFC-nRE-HPC connectivity will correlate with pharmacological antipsychotic response in SCZ subjects. Antipsychotic drugs are usually the elective pharmacological therapy for psychosis. However, it is well known that the response to antipsychotic treatments causes a wide variety of side effects, making it unpredictable and poorly understood. One of the reasons might be that psychotic episodes involve temporal fluctuations in the functional relationships between different brain regions. Therefore, it would be vital to find a potential biomarker for pharmacological antipsychotic response, which, unfortunately, is currently unavailable. A recent systematic review suggested that fMRI imaging functional connectivity (fMRI-FC) may be a predictor of pharmacological antipsychotic response or serve as a biomarker in psychosis (Dominicus et al., 2023).

## 8 Translational relevance of the testable hypothesis

In relation to the testable hypothesis described above, it is relevant to consider their potential translational relevance to identify potential biomarkers, therapeutic implications, and a possible timeline for translating the hypothesis to clinical application.

Concerning the three hypotheses presented here, it is evident that potential biomarkers for PFC-nRE-HPC functional connectivity in SCZ-related cognitive impairments are related to calcium-signaling activity via the CaV3.2 channels, the synchronization of several wave oscillations (γ, θ, β), and fMRI-FC as a predictor of pharmacological antipsychotic response. Moreover, one potential therapeutic implication might be the development of new targeted drugs, such as those that selectively target GABAA receptors containing the α2 subunit, which can inhibit the activity of pyramidal neurons by chandelier neurons in the PFC (Volk and Lewis, 2005). Finally, considering a realistic progression from hypotheses to clinical application, it is essential to note that these hypotheses should be further tested through additional animal studies and complementary computational studies. Moreover, once the hypotheses are proved, pharmaceutical companies should start producing the pharmacological compound, based on studies that confirm its production. Finally, a third stage should be considered, where financed research and clinical trials can be planned and implemented, possibly on a global scale.

## 9 Discussion

### 9.1 Future implications

Here, the potential benefits of integrating *in vivo* and silico theories, approaches, and results were discussed. This integration not only reinforces the strength of the findings but also creates a hypothesis-generating framework in the context of altered PFC-HPC communication and intermediate structures that may contribute to cognitive impairments in psychosis-related conditions. Additionally, to shift the perception of how we approach and treat SCZ and to stimulate further planning for new experimental translational methodologies, we need to implement innovative strategies. These strategies can provide a broad framework for a more nuanced understanding of maladaptive brain communication in psychosis. One particularly intriguing direction involves using the in vivo findings to inform the *in silico* models, which can, in turn, simulate treatment effects in a faster and more cost-efficient manner. For example, Yeh et al. (2024) utilized a non-invasive neuromodulation technique, HD-tDCS, which directly targeted the dlPFC. Upon deliberately targeting the dlPFC, the very weak electrical current resulted in a noticeable reduction in negative symptoms and altered network connectivity. The results of this study have a high potential to inspire promising routes for novel and more effective therapeutic interventions. It also shows promising possibilities in targeting specific actors in specific brain areas. For instance, homologous to optogenetic stimulations at specific frequencies in particular brain structures (Patrono et al., 2023, 2025), HD-tDCS-specific frequency stimulations targeting the dlPFC might have therapeutic value. However, it is essential to note that SCZ animal models are valuable tools for studying the biological underpinnings of the condition and for testing potential therapies. Still, they cannot fully replicate the complex human experience of psychosis, such as hallucinations or delusions, which are impossible to observe in animals directly. While subjective symptoms are challenging to model, cognitive functions (such as perception, attention, and memory) are present in animals. They can be measured using behavioral tests, revealing underlying neural circuits (Arguello et al., 2010). Nonetheless, SCZ animal models are essential for testing the effects of new drugs and interventions, even if they do not fully mirror the human disease. Moreover, genetic models can replicate genetic risk factors for SCZ found in humans, enabling researchers to investigate how specific genetic changes contribute to disease-related behaviors and neurobiological alterations (Forsingdal et al., 2019).

Additionally, it was proposed that there could be a potential for targeted intervention with the help of prefrontal oscillatory slowing, a biomarker of SCZ (Donati et al., 2024). If compared and analyzed together, the studies suggest that if *in silico* models continue to evolve in response to in vivo findings, they could help refine stimulation protocols and even guide the development of more personalized, effective, and accessible treatment strategies. Besides novel therapeutic interventions, another interesting approach within *in silico* studies is the sphere of early intervention, where studies suggest that maladaptive alterations in brain connectivity may often be detected long before the onset of a full-blown psychosis (Øie et al., 2021; Kozhuharova et al., 2021). This highlights the need to identify early biomarkers of SCZ, such as NMDA hypofunction, low GABA availability, and specific altered brain oscillations in particular brain areas (PFC, nRE, vHPC). Therefore, monitoring PFC-HPC connectivity in high-risk individuals may enable the early detection of very subtle changes, proposing a novel avenue for earlier diagnoses and more timely therapeutic support. This is especially crucial for disorders like SCZ, as early detection may delay the onset of chronic symptoms or even mitigate certain worsening aspects of cognitive abilities, making a significant difference. By combining this with an *in silico* approach, researchers can simulate such scenarios, thereby improving the assessment of potential progression risks based on the initial network signatures. Additionally, it could again serve as a helpful tool for a more individually tailored approach (Verma et al., 2023).

This research repeatedly emphasizes the importance of proper functional connectivity, which ensures smooth inter-regional communication and facilitates optimal cognitive functioning. Therefore, treatments that focus on restoring oscillatory synchrony and/or relay functioning (Thorsen et al., 2014) may be the key to significantly improving cognitive symptoms.

### 9.2 Limitations

While there is considerable evidence on the general topic, this research does not establish a definitive causal relationship between the mentioned hypotheses and methodologies. To explain the main point concerning the maladaptive prefrontal-thalamic-hippocampal communication, possibly involved in SCZ-like cognitive inabilities, the current study relied mainly on the “E/I imbalanced ratio hypothesis” as a theoretical background. This hypothesis, by physiological and behavioral means, attempts to explain the reasons for cognitive impairments in SCZ. However, one possible limitation of this study is that the E/I imbalance ratio hypothesis still faces several challenges. Although a few recent human studies have found correlations with the E/I ratio (van Bueren et al., 2023; Sylvester et al., 2025; Sochan et al., 2025), the E/I balance cannot be directly measured in humans, necessitating indirect inferences that may be misleading. Moreover, it is known that normal, healthy brain functions require a delicate E/I. In animals, this balance is known to decrease with the maturation of inhibitory circuitry during healthy development. However, in humans, the normative development of the cortex-wide E/I ratio remains unclear. Nonetheless, a recent study showed that a “whole-cortex E/I ratio” measured using resting-state fMRI confirmed that a lower E/I ratio marker (especially in the association cortex) is linked to better cognitive performance in American and Asian children cohorts (Zhang et al., 2024). To further constrain the E/I imbalanced ratio hypothesis, it has been argued that the brain possesses multiple compensatory mechanisms that can maintain function despite local E/I imbalances, including integrative network interactions and cellular/molecular adaptations. Examples include neuronal and astrocyte adaptations, shifts in network activity (such as increased population sparseness), and modifications to perineuronal nets, which collectively help stabilize neural communication and adapt to changing E/I ratios before a critical threshold is crossed (Papatheodoropoulos, 2025). Regarding the listed limitations of the E/I theoretical framework, readers should note that, although the E/I hypothesis shows promise, it has yet to be fully elucidated. Additionally, translational animal-to-human assumptions should be thoroughly investigated and taken into account.

*In silico* models propose novel and highly intriguing promises in terms of “simulational” capabilities. However, establishing causal relationships between changes in connectivity and cognitive symptoms remains problematic. Recent work demonstrates that functional connectivity measures can be influenced by numerous confounds, including medication effects, head motion, and global signal changes (Kato et al., 2021; Broeders et al., 2024; Doubovikov and Aksenov, 2025). Moreover, alternative therapeutic and pharmacological approaches, along with biomarker predictors, still lack biological validity and reliability. They are novel and not yet commonly applied across various models or long-term settings. Therefore, some debate is needed regarding their current applicability. Yet they should not be disregarded, as they quickly adapt to modern demand and standards. Additionally, individual variability, stage of the disorder, and even comorbidities all play a significant role in the complexity of generalizing these results.

Another limitation of this study is the choice of the review process. It has been noticed that a more “systematic review methodology” process, which could have included inclusion/exclusion criteria for gathering findings, a quality assessment of included studies, and statistical synthesis methods, might have provided a deeper understanding of the topic addressed here. However, the primary aim of this study was to summarize recent findings regarding specific aspects of SCZ-like cognitive impairments and to produce a narrative review, primarily compiling findings from our previous studies and those of others in the field.

Despite the limitations, this research uncovered several significant findings that helped establish a baseline for future investigations. It is beneficial to focus on the future expansion of the computational simulation approach, exploring the entire PFC-HPC connectivity loop (PFC-nRE-HPC), as this may yield interesting findings. Moreover, a longitudinal approach could be employed to ensure the proper validation of certain *in silico* models, particularly with the aid of neuroimaging and real-life modulation. However, focusing on how dynamic functional connectivity evolves and changes throughout the disorder would be crucial (Sendi et al., 2021).

## 10 Conclusion

The main goal of this study was to draw attention to the maladaptive PFC-nRE-HPC communication in psychosis-induced cognitive inabilities. We gathered information from *in vivo* and *in silico* studies to propose a generating-hypothesis framework that could be further used to develop new translational and multimodal approaches to investigate the specific roles of individual actors involved in psychosis-induced cognitive impairments. A large body of literature supports the hypothesis that higher-order cognitive deficits might be affected by the dysfunctional PFC-HPC communication in SCZ. Animal studies and human studies, although employing different approaches and methodologies, have demonstrated maladaptive oscillatory activities in brain waves, which are presumably linked to the altered cognitive functions observed in SCZ. It is essential to note that humans and other animal species used as models differ in brain organization due to variations in cortical area, size, complexity, and connectivity, as well as differences in the timing of neuron generation and structural complexity during development (Eichert et al., 2020). Therefore, it is not possible to draw a straightforward comparison between human and animal studies; however, it is more productive to use them to create a new framework for building new hypotheses. Conversely, *in silico* studies, which are based on computational evaluations and thus highly speculative, may be used for hypothetical simulations. In this context, multiple translational methodologies may help create new evidence. The shift in the way SCZ is viewed may inspire more research and offer promising possibilities for refining therapeutic strategies and identifying biomarkers for early detection and intervention.
